# Determination of an Objective Criterion for the Assessment of the Feasibility of an Instrumented Indentation Test on Rough Surfaces

**DOI:** 10.3390/ma13071589

**Published:** 2020-03-30

**Authors:** Julie Marteau, Abdeljalil Jourani, Maxence Bigerelle

**Affiliations:** 1 Laboratoire Roberval FRE-CNRS 2012, Centre de Recherches de Royallieu, Sorbonne Université, Université de Technologie de Compiègne, 60200 Compiègne, France; abdeljalil.jourani@utc.fr; 2 Laboratoire d’Automatique, de Mécanique et d’Informatique industrielle et Humaine (LAMIH) UMR-CNRS 8201, Université Polytechnique des Hauts de France, Le Mont Houy, 59313 Valenciennes, France; maxence.bigerelle@uphf.fr

**Keywords:** hardness, indentation, roughness, surface topography

## Abstract

The influence of roughness on the results of indentation testing was investigated using a semianalytical model. This model used simulated surfaces that were described using three standard roughness parameters: the root-mean-square deviation S_q_, the wavelength (or cut-off of Gaussian high-pass filter), and the fractal dimension. It was shown that S_q_ had the largest effect on the determination of the macrohardness, while the surface wavelength and fractal dimension had negligible effects at the scale of investigation. The error of determination of the macrohardness rose with the increase of the ratio S_q_/h_max_ where h_max_ was the maximum indentation depth: S_q_/h_max_ ratios lower than 0.02 were required to obtain a systematic error of the macrohardness lower than 5%, whatever the examined material mechanical properties (in elasticity and plasticity).

## 1. Introduction

Instrumented indentation testing is a widely used technique for the determination of mechanical properties. However, smooth surfaces are required to avoid the introduction of uncertainties in the determination of the contact area [1]. The need for smooth surfaces can be an issue for different reasons:

(i) If the investigation of the material properties of a given component is required, then surface preparation may not be wished for to avoid affecting the surface properties or to avoid deteriorating the component.
(ii) It is impossible to have a surface free of any roughness: whatever the surface preparation, there is always residual roughness that may impact the very small scales.

The impact of roughness on the indentation results was investigated by several authors. Walter et al. [2] used finite element simulation to examine the influence of roughness on the scattering of indentation curves and on the computation of Young’s modulus using the Oliver–Pharr method [3]. Similarly, Li et al. [4] analyzed the effect of roughness on the determination of hardness using finite element simulation. Wei et al. [5] developed a new model for the examination of microcontact to assess the impact of surface roughness on the contact force and the contact area. Qasmi et al. [6] tested several materials presenting different surface morphologies to quantify the influence of the value of the root-mean-square roughness parameter on the computed Young’s modulus and hardness values. Similarly, Jiang et al. [7] coupled finite element methods with atomic methods to assess the effect of roughness on the computation of Young’s modulus and hardness on thin films of single-crystal copper. They found that neglecting roughness effects required indentation depths much larger than the characteristic size of surface roughness. According to the International Standard ISO 14577-1 [8], the arithmetic average roughness R_a_ should be less than 5% of the maximum indentation depth. Thus, some experimenters use larger loads to rule out roughness influence: as an example, Wai et al. [9] showed that higher loads were needed in order to test rougher polymer samples. Other experimenters developed methods in order to determine mechanical properties despite non-negligible roughness. Arivuoli et al. [10] measured profiles before and after indentation to measure hardness at shallow depths of indentation. Kim et al. [11] proposed to analytically separate the work required to flatten the surface from the one required to deform the flattened surface. Souza et al. [12] corrected the tip-surface contact using a methodology based on the contact stiffness observation. Chicot et al. [13] proposed a methodology based on the correction of the loading curve using Proportional Specimen Resistance [14]. As for Bobji et al. [15], they presented a method that separated roughness from material property gradient but this method can only be used when respecting a defined geometry and when the indentation depth is larger than thrice the root-mean-square roughness.

The aim of this paper is to provide an objective criterion for assessing the feasibility of an instrumented indentation test on rough surfaces. This criterion will reflect the disturbances caused by the material morphology and will indicate if the results of the indentation test are appropriate for the determination of mechanical properties.

## 2. Materials and Methods

To assess the effect of roughness on the indentation curves and the resulting macrohardness, a semianalytical model of contact was applied on surfaces obtained with the Weierstrass function. This choice can be explained by several elements:

- Surface roughness being stochastic, a large number of numerical simulations were required to obtain representative results. A semianalytical model was used to simulate numerous indentation tests on rough surfaces to get a high number of computations with low computation time.
- Experimentally, it is impossible to change the morphology of a surface without impacting the subsurface properties. The use of the Weierstrass function was preferred to examine the influence of the characteristic parameters of the surface (e.g., amplitude, wavelength) independently of any material property changes. Inversely, the characteristic parameters of the surface can be modified without altering the mechanical properties of the surface.
- This methodology avoided the introduction of any measurement noises or experimental artifacts.

As for the resulting macrohardness, it was computed using a methodology based on the minimization of the discrepancies between the shapes of the experimental loading curves and the shapes of the curves predicted by Kick’s law [16,17].

### 2.1. Surface Characteristics

The modified Weierstrass function was used to simulate the surfaces. Three-dimensional fractal Weierstrass surfaces *W* were defined as

(1) $$W\left(x,y\right)=A\;\overset{\infty }{\underset{n_{1}=0}{\sum }}\overset{\infty }{\underset{n_{2}=0}{\sum }}a_{n_{1}n_{2}}\frac{\gamma^{\frac{n_{1}+n_{2}}{2}}}{{\left[\gamma^{2n_{1}}+\gamma^{2n_{2}}\right]}^{\frac{H+1}{2}}}\cos\left(2{\pi \gamma }^{n_{1}}x+\phi_{n_{1}n_{2}}\right)\cos\left(2{\pi \gamma }^{n_{2}}y+\Psi_{n_{1}n_{2}}\right),$$

where *A* is a scaling coefficient, *a*_*n*1,*n*2_ is a normal random number, *H* is the Hölder exponent (H belongs to [0,1]), and γ is the frequency of the function with γ > 1. *ϕ*_*n*1,*n*2_ and *ψ*_*n*1,*n*2_ are stochastic phases of uniform density defined in [0,2π].

The created surfaces were characterized by three parameters:

- The amplitude described by the root-mean-square deviation S_q_, as described in [18]. The following values were tested: 0.001, 0.002, 0.0039, 0.0078, 0.0156, 0.0313, 0.0625, 0.125, 0.25, 0.5, 1, 2, 4, and 8 µm. It should be noted that very small values of S_q_ are only used as asymptotic values.
- The wavelength. The following values were used: 0.2496, 0.4992, 1.0016, 2, 4, 8, 16, 32, 64, 128, and 256 µm. To be consistent with the standard, the wavelength was not defined by series truncation of Equation (5) but was obtained with the cut-off frequency of Gaussian high-pass filtering as defined in [19].
- Fractal dimension. Values ranging from 2 to 3 using an increment of 0.1 were used.

**Figure 1:**
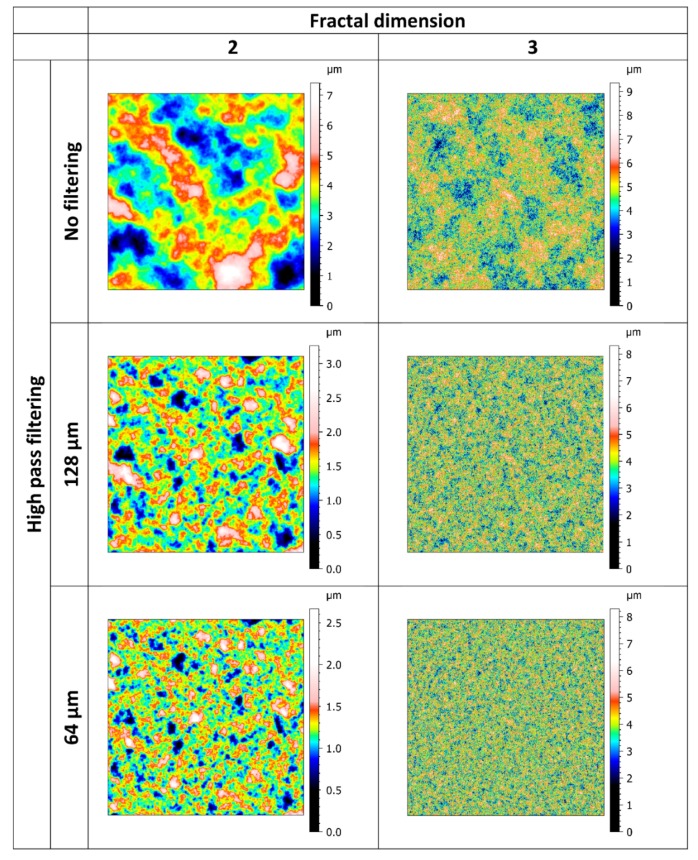
Examples of Weierstrass surfaces (1024 × 1024 µm^2^) with S_q_ equal to 0.5 µm, fractal dimensions equal to two or three, and no filtering or high-pass filtering with different wavelengths.

The effect of discretization was tested using different mesh sizes: 64 × 64, 128 × 128, 256 × 256, 512 × 512, 1024 × 1024, 2048 × 2048, and 4096 × 4096. All the produced surfaces were based on high resolution: no truncations were made on the Weierstrass function. Lower resolutions were obtained with an interpolation based on the spline function. Examples of Weierstrass surfaces are shown in Figure 1.

### 2.2. Description of the Semianalytical Model Used for Simulating Indentation Testing

The semianalytical model used for simulating indentation testing assumed that a rough and hard surface was in contact with a perfectly smooth rigid indenter having a Berkovich tip. Contacts were assumed to be local: they took place at the summits of the surface. The analysis of local summit geometry was used to numerically investigate the behavior of each asperity. Roughness was described as a series of cones having hemispherical end tips. The asperities were described using a distribution of contact angles. The semianalytical model was based on Johnson’s equations describing elastic-plastic indentation with a conical indenter [20]. According to Johnson’s equations, the mean pressure *p_jm_* undergone by a given asperity was

(2) $$p_{jm}=\frac{2}{3}R_{e}\left(1+\ln\left(\frac{E\;tan\theta_{j}}{3R_{e}}\right)\right)$$

where *R_e_* is the elastic limit, *E* is Young’s modulus of the material, and *θ* is the attack angle. In this study, either an elastic deformation or a fully plastic deformation was assumed for the asperity. Elastic interactions between asperities were neglected. The full deformation led to

(3) $$p_{m}\;\sim \;3R_{e}\;\sim \;H_{th}$$

where *H_th_* is the macrohardness of the material.

Computing was performed using elliptic local areas of contact with semiaxes *a_j_* and *b_j_*. This choice led to the elastoplastic deformation of the asperities being neglected. The local areas of contact were divided into N elements *c_ji_* (*i* = 1, …, N). Local pressure on a given asperity *j* was distributed as follows:

(4) $$p_{j}\left(x_{i},y_{i}\right)=\frac{3}{2}p_{jm}{\left(1-{\left(\frac{x_{i}}{a_{j}}\right)}^{2}-{\left(\frac{y_{i}}{b_{j}}\right)}^{2}\right)}^{1/2}$$

The corresponding normal force *F_j_* applied on an asperity was

(5) $$F_{j}=\overset{}{\underset{i}{\sum }}c_{ji}p_{i}$$

The load applied to the summits of a rough surface was equal to the sum of the normal forces *F_j_*. All the details of the numerical model can be found in [21].

**Figure 2:**
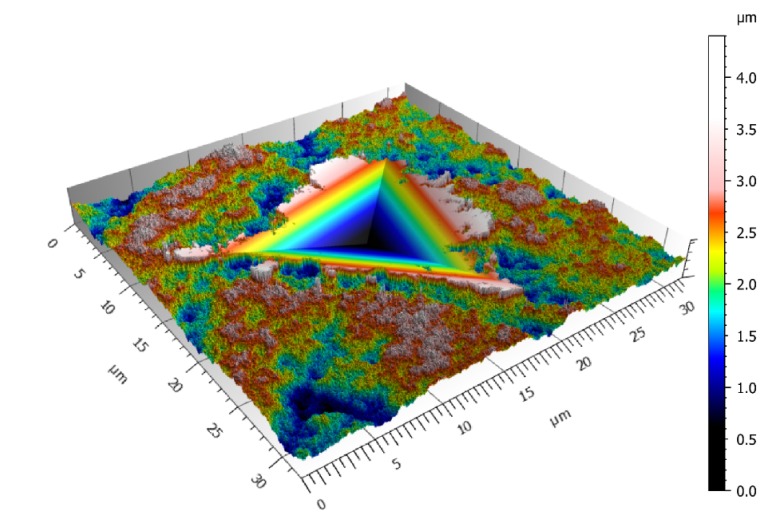
Example of the indentation of a Weierstrass surface with a S_q_ of 0.5 µm, a fractal dimension of 2.5, and no-high-pass filtering. The indenter is not shown but is still in contact with the surface.

The maximum indentation depth for the numerical simulations was equal to 3 µm. For most of the computations, the material Young’s modulus E was equal to 110 GPa and the elastic limit R_e_ was set to 1050 MPa. Poisson’s coefficient was set to 0.3. The plastic behavior was assumed to be fully plastic, and Tresca’s criterion was used. To integrate the stochastic aspect of the surface morphology, at least one hundred computations were performed for each studied case. An example of the indentation of a Weierstrass surface with a S_q_ of 0.5 µm, a fractal dimension of 2.5, and no-high-pass filtering is given in Figure 2.

### 2.3. Hardness Computation Method

As non-negligible topography causes the scatter of the indentation curves; macrohardness was computed using a methodology first presented in [16]. In short, the loading part of the indentation curves was described using Kick’s law [17,22] (and not Bernhardt’s law as in [16,23] as the Indentation Size Effect was assumed to be nil in the present article). The equation given by Kick’s law was rewritten using the contact depth *h_c_* used by Oliver and Pharr in [3] and the macrohardness *H*_0_. Then, it was assumed that there was a discrepancy between Kick’s law and the actual shape of the computed loading curves, called Δ*h_c_*. Finally, the following minimization was performed on all the computed loading curves in order to identify *H*_0_

(6) $$\underset{H_{0},\Delta h_{1}\ldots \;\Delta h_{n}}{\min}\overset{n}{\underset{i=1}{\sum }}\overset{p_{i}}{\underset{j=1}{\sum }}{\left[P_{i,j}-\alpha \left(H_{0}h_{c,j}{}^{2}+\left(2H_{0}\Delta h_{c,i}\right)h_{c,j}+H_{0}\Delta h_{c,i}{}^{2}\right)\right]}^{2}$$

where index *j* refers to a point belonging to curve *i* and *α* is a constant linked with the indenter geometry.

## 3. Results

**Figure 3:**
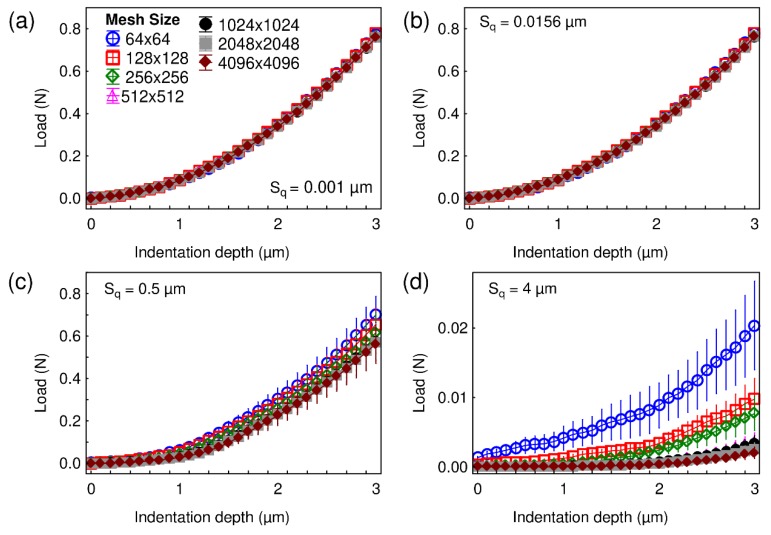
Load-indentation depth curves obtained for different mesh sizes with a root-mean-square roughness S_q_ equal to (**a**) 0.001 µm, (**b**) 0.0156 µm, (**c**) 0.5 µm, and (**d**) 4 µm. The wavelength and fractal dimension of the tested surfaces are, respectively, set to 32 µm and 2.5.

Before studying the influence of roughness on the determination of hardness, indentation curves were computed for different values of the root-mean-square roughness S_q_ using fixed wavelength and fractal dimension values. Figure 3 shows the loading parts of the indentation curves obtained with the semianalytical model for a maximum indentation depth equal to 3 µm. The wavelength and fractal dimension of the tested surfaces were, respectively, set to 32 µm and 2.5, while the root-mean-square roughness S_q_ was equal to (a) 0.001 µm, (b) 0.0156 µm, (c) 0.5 µm, and (d) 4 µm. Different mesh sizes were tested for each examined case. For S_q_ values of 0.001 µm and 0.0156 µm, it can be seen that all the loading curves are superimposed, whatever the tested mesh sizes, and very low standard deviations are observed. For S_q_ equal to or larger than 0.5 µm, there is a change of trend: the loading curves are superimposed for small indentation depths, and then the curves obtained with lower mesh sizes tend to show larger loads than the curves computed with larger mesh sizes, for a given indentation depth. For S_q_ equal to 0.5 µm, the standard deviations of all the curves overlap. On the contrary, when S_q_ is equal to 4 µm, the curves obtained with mesh dimensions equal to 64 × 64, 128 × 128, and 256 × 256 show different trends than the ones obtained with mesh sizes equal to or larger than 512 × 512. Moreover, these curves have very low load values compared to the other tested S_q_ values for similar indentation depths. It seems that the surface roughness is too large compared to the indentation depth. The surface asperities only began to flatten, thus giving low load values. To underline the importance of the scale and as the examined contact case has selfsimilar geometry, dimensionless results were computed for the following figures, i.e., the results are expressed using the ratio S_q_/h_max_, where h_max_ is the maximum indentation depth.

**Figure 4:**
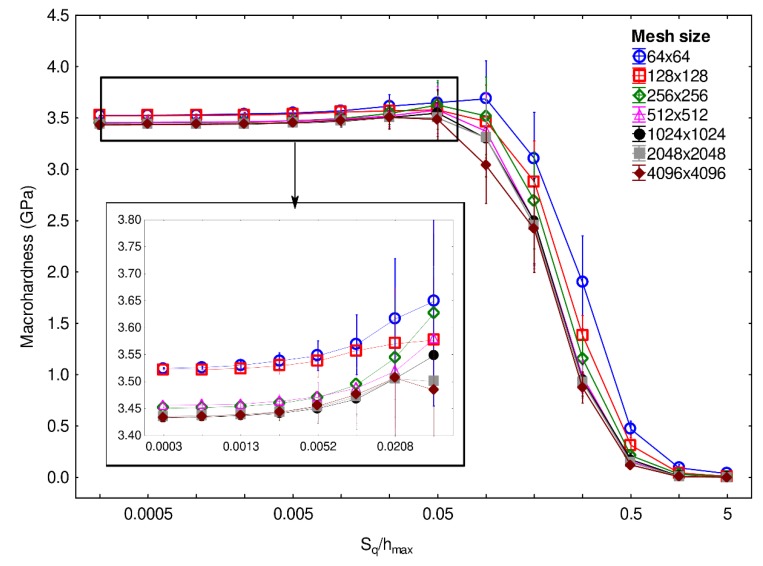
Macrohardness as a function of the ratio S_q_/h_max_, which is the root-mean-square roughness divided by the maximum indentation depth, computed with different mesh sizes. The wavelength and fractal dimension of the tested surfaces are, respectively, set to 32 µm and 2.5.

The methodology introduced in Section 2.3 was then applied to all the computed loading curves to determine the macrohardness for different values of mesh sizes and root-mean-square roughness S_q_. Figure 4 illustrates the computed macrohardness results as a function of S_q_ divided by the maximum indentation depth h_max_, for different mesh sizes. The wavelength and fractal dimension of the tested surfaces were, respectively, set to 32 µm and 2.5. For S_q_/h_max_ ratios equal to or lower than 0.04, the computed macrohardness values are between 3.4 and 3.7 GPa and are thus close together, even if globally larger macrohardness values are identified for lower mesh sizes. Then, for S_q_/h_max_ ratios greater than 0.04, whatever the mesh size, the macrohardness values significantly decrease and reach zero for ratios equal to or larger than approximately 1.5. According to these results, it seems that, for S_q_/h_max_ ratios greater than 0.04, indentation testing does not enable the macrohardness of the examined material to be accurately determined.

**Figure 5:**
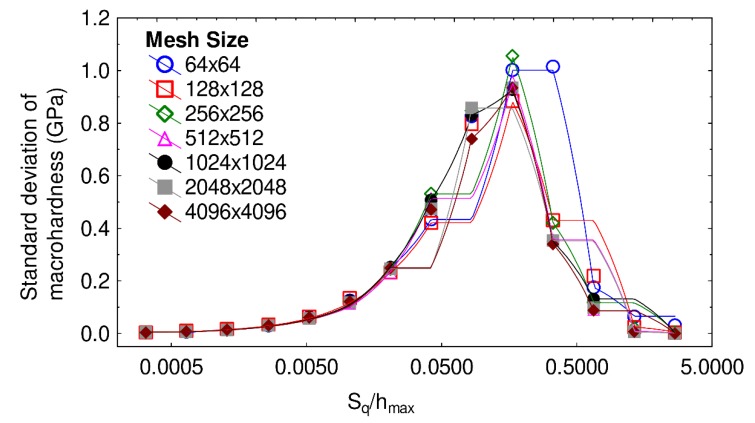
Standard error of macrohardness as a function of the ratio S_q_/h_max_, for different mesh sizes. The wavelength and fractal dimension of the tested surfaces are, respectively, set to 32 µm and 2.5.

In order to confirm these results, the variation of the standard deviation of the macrohardness was examined as a function of the ratio S_q_/h_max_ for different mesh sizes in Figure 5, using the same wavelength and fractal dimension as in Figure 4. For a ratio S_q_/h_max_ larger than 0.04, the macrohardness standard deviation becomes non-negligible as it is larger than 0.5 GPa. The same transition zone, as in Figure 4, is thus identified. The macrohardness standard deviation rises to a maximum approximately equal to 1 GPa for a ratio S_q_/h_max_ around 0.2, whatever the mesh size. The lower standard deviations of macrohardness observed for ratios greater than 0.2 are correlated with the decrease of macrohardness observed in the previous graph. Hereafter, the mesh size is fixed and is equal to 256 × 256 as it gives representative results and enables quick computation to be performed.

**Figure 6:**
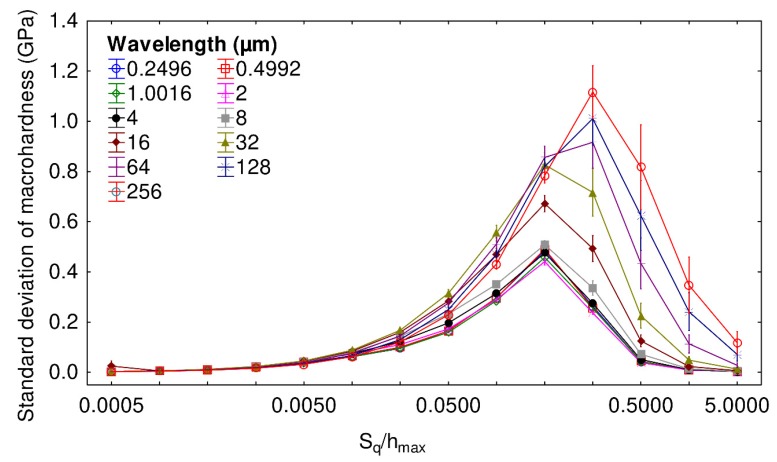
Standard deviation of macrohardness as a function of the ratio S_q_/h_max_ for different values of wavelengths. The mesh size and fractal dimension of the tested surfaces are, respectively, set to 256 × 256 and 2.5.

Wavelength influence is now examined. Figure 6 illustrates the variations of the macrohardness standard deviation plotted as a function of the ratio S_q_/h_max_ for different wavelengths. A trend similar to the one obtained for the previous figure is observed. Whatever the observed wavelength, the macrohardness standard deviation first increases and then decreases as a function of the ratio S_q_/h_max_. The identified maxima are slightly different depending on the wavelength: for wavelengths equal to 32 µm or lower, the maximum is reached when S_q_/h_max_ is equal to 0.2, while it is reached for a ratio equal to 0.3 for larger wavelengths. For a ratio S_q_/h_max_ equal to 0.04, very similar results are obtained whatever the wavelength as the macrohardness standard deviation is between 0.15 and 0.3 GPa. Thus, globally, the wavelength has a small impact on the standard deviation of hardness, compared to the root-mean-square roughness S_q,_ for ratios S_q_/h_max_ lower than 0.04.

In order to observe the variation of the macrohardness as a function of S_q_, another indicator is examined: the macrohardness absolute error, which is computed as follows:

(7) $$E_{rr\_abs}=\left(\frac{\left|H_{th}-H_{0}\right|}{H_{th}}\right)\times 100$$

where *E_rr_abs_* is the microhardness absolute error, *H_th_* is the hardness value used in the simulation (3.5 GPa), and *H*_0_ is the value computed for the macrohardness using the methodology described in Section 2.3.

**Figure 7:**
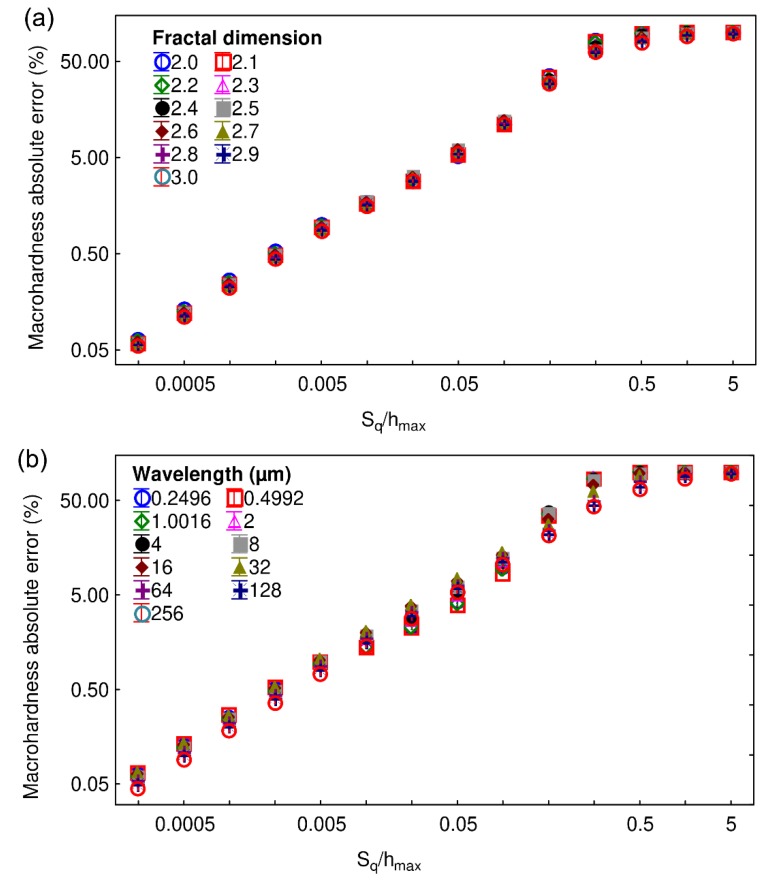
The macrohardness absolute error as a function of S_q_ is computed using (**a**) different fractal dimensions and a fixed wavelength equal to 32 µm and (**b**) different wavelengths with a fractal dimension set to 2.5. The mesh size is 256 × 256.

Figure 7 shows the computation of the macrohardness absolute error as a function of the ratio S_q_/h_max_ for (a) different fractal values with a fixed wavelength equal to 32 µm and (b) for different wavelength values and a fixed fractal dimension equal to 2.5. Whatever the observed graph, the standard deviations are very small. Both graphs also show the same trend as a function of the increase of the ratio S_q_/h_max_: the macrohardness absolute error increases until it reaches a plateau for a value of S_q_/h_max_ around 0.3. The first part of the curve follows a power law representing the error made in the computation of macrohardness caused by roughness: this is a systematic error. For a S_q_/h_max_ ratio equal to 0.04, the macrohardness absolute error is equal to 5%, whatever the tested wavelength or fractal dimension.

**Figure 8:**
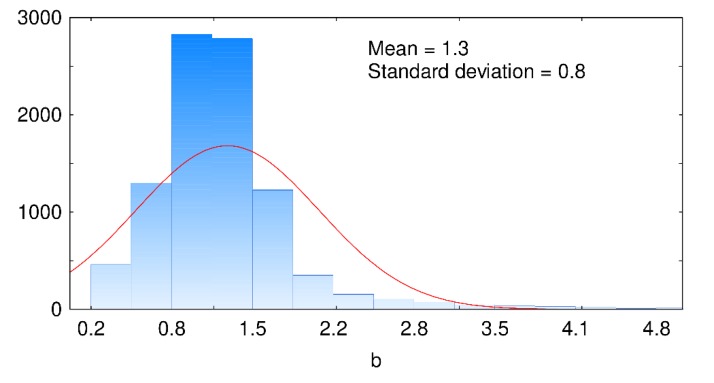
Distribution of coefficient b.

If one assumes that the macrohardness absolute error follows a power law having an exponent called b for values of S_q_/h_max_ lower than 0.3 and if one tests this law for all the fractal dimensions and wavelength presented in Figure 7, then the histogram shown in Figure 8 is obtained. The average value of coefficient b is equal to 1.3, while its standard deviation is equal to 0.8. The identified coefficient is neither dependent on the fractal dimension values nor on the wavelength values. This identification means that the error committed on macrohardness determination increases by a power of 1.3 with the increase of the ratio S_q_/h_max_.

In order to check if the identified relationships between the macrohardness value and the root-mean-square roughness S_q_ are not dependent on the studied material, different values of Young’s modulus E and elastic limit R_e_ are tested using the semianalytical model. As the macrohardness values are directly linked with the tested elastic limits, the macrohardness coefficient of variation is computed in order to assess the effect of the material properties. The macrohardness coefficient of variation (CV) is defined as follows:

(8) $$CV=\frac{\sigma }{\mu }$$

where *σ* and *µ* are the standard deviation and the mean value of the macrohardness, respectively. It should be noted that the computation of the hardness coefficient of variation requires a mean value different from zero and the use of at least two hardness values. Both conditions are respected in the examined cases.

**Figure 9:**
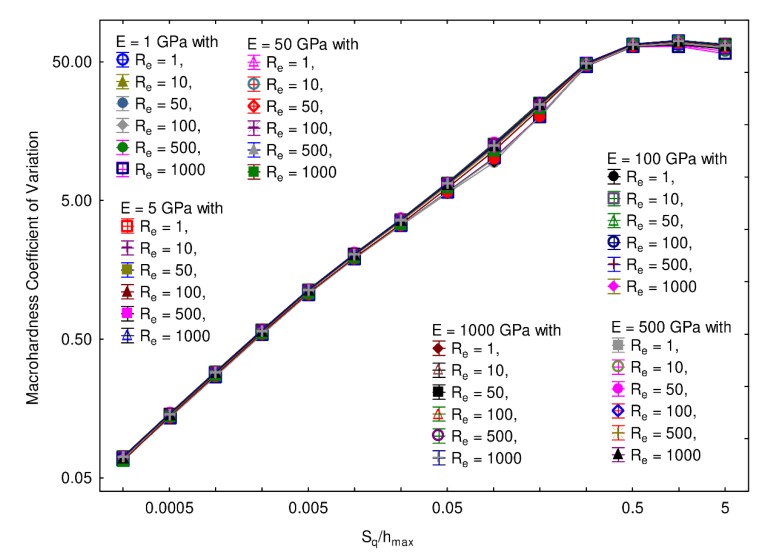
Coefficient of variation of the macrohardness as a function of the ratio S_q_/h_max_ for different values of Young’s modulus E and elastic limit R_e_. The mesh size, wavelength, and fractal dimension were, respectively, set to 256 × 256, 32 µm, and 2.5.

Figure 9 shows the macrohardness coefficient of variation computed as a function of the ratio S_q_/h_max_ for values of Young’s modulus E comprised between 1 and 500 GPa, and values of elastic limit R_e_ between 1 and 1000 MPa. The computations made with different values of E and R_e_ give trends that are nearly identical. Once again, for S_q_/h_max_ ratios lower than 0.3, a power law can be identified.

**Figure 10:**
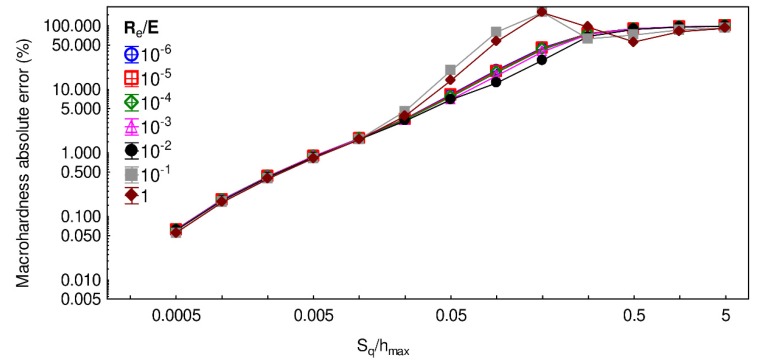
Macrohardness absolute error as a function of the ratio S_q_/h_max_ for different values of R_e_/E ratio. The mesh size, wavelength, and fractal dimension were, respectively, set to 256 × 256, 32 µm, and 2.5.

In the literature, the impact of mechanical properties on indentation test results is often assessed by observing the ratios R_e_/E, e.g., [24,25]. The computation of the macrohardness absolute error as a function of the ratio S_q_/h_max_ for different values of R_e_/E is illustrated in Figure 10. Globally, the macrohardness absolute error follows the same trend whatever the tested R_e_/E ratio for S_q_/h_max_ ratios lower than 0.02 and larger than 1.3. Within this interval, low values R_e_/E ratio show larger macrohardness absolute error values. According to this graph, having a systematic error below 5% requires an indentation depth that is approximately fifty times larger than S_q_, whatever the examined material.

**Figure 11:**
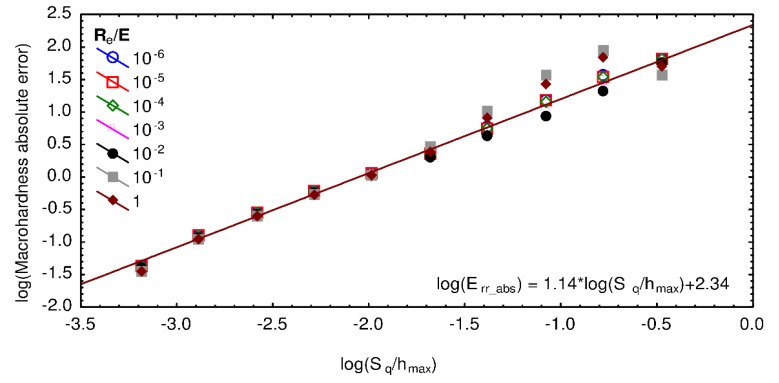
Logarithm of the macrohardness absolute error as a function of the logarithm of the ratio S_q_/h_max_ for different values of R_e_/E ratio. The mesh size, wavelength, and fractal dimension were, respectively, set to 256 × 256, 32 µm, and 2.5.

Using logarithmic values for both axes and only showing S_q_/h_max_ ratios lower than 1, Figure 11 is obtained. Through the use of linear regression analysis, the following relationship is identified

(9) $$\log_{10}Err\_abs=1.14\log_{10}\frac{S_{q}}{h_{max}}+2.34.$$

The previous equation is valid for S_q_/h_max_ ratios lower than 0.01, whatever the mechanical properties or for values of S_q_/h_max_ ratios between 0.01 and 0.5 for R_e_/E ratios lower than 10^−2^.

## 4. Conclusions

Roughness effects on the indentation test results were examined using a semianalytical model. The use of a semianalytical model enabled a large number of calculations with low computation time to be obtained and is thus ideal for examining the error made on the computation of the macrohardness when using a methodology based on a minimization of the gaps between the shapes of the experimental loading curves and the ones predicted by Kick’s law.

Using both the semianalytical model and the methodology for computing the macrohardness, it was first found that the surface amplitude described by the root-mean-square deviation S_q_ had the largest influence on the macrohardness results. The two other parameters used to describe the surface. i.e., the surface wavelength and fractal dimension, had negligible effects on the macrohardness determination at the scale of investigation (i.e., when focusing on low errors on the macroharness computation).

Then, it was shown that the error made on the macrohardness determination increased as a function of the ratio S_q_/h_max,_ where h_max_ is the maximum indentation depth. It followed a power law having an exponent equal to 1.3 for maximum indentation depths that are larger than thrice the root-mean-square deviation S_q_. This law was dependent neither on wavelength variations nor on fractal dimension variations.

Finally, the error committed on the computed macrohardness was shown to be independent of the mechanical properties values for S_q_/h_max_ ratios lower than 0.01. Then, for S_q_/h_max_ ratios between 0.01 and 0.5, the error was shown to be independent of the mechanical properties for R_e_/E ratios smaller than 10^−2^. Finally, it was shown that in order to obtain a systematic error of the macrohardness lower than 5%, whatever the examined material mechanical properties, a S_q_/h_max_ ratio lower than 0.02 was required. Future work will focus on the building of surfaces with controlled roughness features to further investigate and test the effect of roughness on indentation testing.

## Nomenclature

α	Constant linked to indenter geometry
a_j_, b_j_	Semiaxes of ellipse
b	Power-law exponent
CV	Coefficient of variation
Δh_c_	Discrepancy between Kick’s law and computed indentation curve
E	Young’s modulus
Err_abs	Macrohardness absolute error
F_j_	Normal force
h_c_	Contact depth
h_max_	Maximum indentation depth
H	Hölder exponent
H_th_	Macrohardness of the material
H_0_	Macrohardness computed with curve shifting methodology
p_j_	Local pressure
p_jm_	Mean pressure undergone by a given asperity j
P	Indentation load
R_e_	Elastic limit
S_q_	Root-mean-square deviation
θ	Angle of attack
